# Recruitment of Neutrophils Mediated by Vγ2 γδ T Cells Deteriorates Liver Fibrosis Induced by Schistosoma japonicum Infection in C57BL/6 Mice

**DOI:** 10.1128/IAI.01020-16

**Published:** 2017-07-19

**Authors:** Li Zheng, Yuan Hu, Yanjuan Wang, Xibao Huang, Yuxin Xu, Yujuan Shen, Jianping Cao

**Affiliations:** a National Institute of Parasitic Diseases, Chinese Center for Disease Control and Prevention, Key Laboratory of Parasite and Vector Biology, Ministry of Health, China, National Center for International Research on Tropical Diseases, China, and WHO Collaborating Center for Tropical Diseases, Shanghai, China; b Hubei Provincial Center for Disease Control and Prevention, Hubei Provincial Academy of Preventive Medicine, Wuhan, China; University of South Florida

**Keywords:** γδ T cell, neutrophil, mice, Schistosoma japonicum, liver fibrosis, IL-17A

## Abstract

Conventional adaptive T cell responses contribute to the pathogenesis of Schistosoma japonicum infection, leading to liver fibrosis. However, the role of gamma-delta (γδ) T cells in this disease is less clear. γδ T cells are known to secrete interleukin-17 (IL-17) in response to infection, exerting either protective or pathogenic functions. In the present study, mice infected with S. japonicum are used to characterize the role of γδ T cells. Combined with the infection of S. japonicum, an extremely significant increase in the percentage of neutrophils in the CD45^+^ cells was detected (from approximately 2.45% to 46.10% in blood and from 0.18% to 7.34% in spleen). Further analysis identified two different γδ T cell subsets that have different functions in the formation of granulomas in S. japonicum-infected mice. The Vγ1 T cells secrete gamma interferon (IFN-γ) only, while the Vγ2 T cells secrete both IL-17A and IFN-γ. Both subtypes lose the ability to secrete cytokine during the late stage of infection (12 weeks postinfection). When we depleted the Vγ2 T cells in infected mice, the percentage of neutrophils in blood and spleen decreased significantly, the liver fibrosis in the granulomas was reduced, and the level of IL-17A in the serum decreased (*P* < 0.05). These results suggest that during S. japonicum infection, Vγ2 T cells can recruit neutrophils and aggravate liver fibrosis by secreting IL-17A. This is the first report that a subset of γδ T cells plays a partial role in the pathological process of schistosome infection.

## INTRODUCTION

Schistosomiasis is endemic to many tropical and subtropical regions globally, including China and the Philippines, where Schistosoma japonicum is endemic (1). In total, schistosomiasis affects more than 200 million people (2). Progression of schistosomiasis from the time of egg deposition through the development of mature granulomas in the liver and hepatic fibrosis has been associated with distinct temporal gene expression patterns (3). Based on these patterns, neutrophils may play a significant role in determining the outcome of S. japonicum infection. The recruitment of neutrophils to the liver has been associated with the development of fibrosis in other chronic liver diseases (4–6), suggesting they could contribute to fibrosis in schistosomiasis.

Interleukin-17 (IL-17) has been linked to neutrophil infiltration in the liver during schistosomiasis caused by S. japonicum (7, 8) and is related to the development of liver fibrosis (1). Of the cells known to secrete IL-17, gamma-delta (γδ) T cells play a crucial role in the immune system. These cells represent a small population of the overall T lymphocytes (0.5% to 5%) and are known to be the first line of host defense against pathogens, including those causing malaria and tuberculosis (9). γδ T cells have been shown to secrete Th1 (gamma interferon [IFN-γ] and tumor necrosis factor alpha [TNF-α]), Th2 (IL-4 and IL-10), and antigen-presenting cells like cytokines (IL-12) under different circumstances. They have also been shown to produce IFN-γ, IL-17, IL-4, IL-5, IL-10, IL-13, transforming growth factor beta (TGF-β), and granulocyte-macrophage colony-stimulating factor (GM-CSF). There are several distinct subsets of γδ T cells that have different functions in inflammation and autoimmunity (10, 11). *Ex vivo*, γδ T cells in the intestinal intraepithelial lymphocyte (IEL) population can be activated with anti-T cell receptor (TCR) antibody, and the Vγ2 T cells in particular produce IL-17 (12).

It is unclear whether γδ T cells contribute to liver fibrosis during S. japonicum infection (13, 14). In one study in which γδ^−\−^ mice were infected with S. japonicum, there was no evidence that γδ T cells were required for granuloma formation in the liver (15). Moreover, when RAG^−/−^ mice were infected, granulomas still developed (16). However, another study found that IL-17 secretion by liver lymphocytes was significantly enhanced by S. japonicum infection (1). γδ T cells have been shown to recruit neutrophils by secreting IL-17A in models such as breast cancer and Listeria monocytogenes infection (17). Sporadic reports address the behavior of γδ T cells in S. japonicum-infected mice, but they do not address interaction with neutrophils (18–20). Therefore, to better understand the role of γδ T cells in S. japonicum infection, we used the C57BL/6 mouse infection model to characterize the cytokine profile and effects of γδ T cell function on neutrophils.

## RESULTS

### The percentages of neutrophils in the blood and spleen were increased following S. japonicum infection.

**FIG 1:**
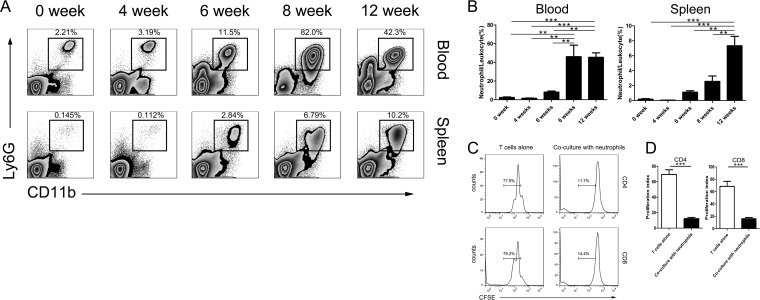
Percentage of neutrophils in the white blood cell population increased postinfection. (A) Representative flow cytometry plots are shown to describe the increase in neutrophils in the blood and spleen following S. japonicum infection. Neutrophils were defined as CD45^+^ CD11b^+^ Ly6g^+^ Ly6c^+^ F4/80^−^ and are shown as a percentage of the total white blood cell (CD45^+^) population. (B) Summary graphs showing the increase in neutrophils as a percentage of total white blood cells for 4 animals following infection with S. japonicum. Animals were monitored longitudinally for up to 12 weeks. **, *P* < 0.01; ***, *P* < 0.001 (by one-way ANOVA with Tukey's *post hoc* test). (C) CD3^+^ T cells were labeled with CFSE and then cultured alone or with neutrophils from S. japonicum-infected mice. Proliferation was measured as CFSE dilution after 48 h in culture. Representative CFSE dilution plots showing T cell proliferation are shown. (D) Summary graphs showing the reduction in T cell proliferation following coculture with neutrophils. Three or four samples per group were used. ***, *P* < 0.001 (by Student's *t* test).

To begin, we first characterized the expansion of neutrophils in the peripheral blood and spleen of S. japonicum-infected C57BL/6 mice. Following infection, the percentage of neutrophils (CD45^+^ CD11b^+^ Ly6g^+^ Ly6c^+^ F4/80^−^) in the white blood cell (CD45^+^) population remained low in the peripheral blood from 0 (2.45%) to 4 (1.57%) weeks postinfection, and then it increased beginning 6 weeks (8.18%) and peaked 8 weeks (46.1%) postinfection, still remaining high at the 12th week (45.5%). The difference between the groups was significant (*P* < 0.001). The differences between the uninfected and 8-week groups (*P* < 0.01), uninfected and 12-week groups (*P* < 0.001), 4- and 8-week groups (*P* < 0.01), 4- and 12-week groups (*P* < 0.001), 6- and 8-week groups (*P* < 0.01), and 6- and 12-week groups (*P* < 0.01) were significant. Similar trends were observed in the spleen, which were 0.18%, 0.06%, 1.12%, 2.56%, and 7.34%, respectively. The differences between the groups was significant (*P* value of <0.001 by one-way analysis of variance [ANOVA]). The difference between uninfected and 12-week groups (*P* < 0.001), 4- and 12-week groups (*P* < 0.001), 6- and 12-week groups (*P* < 0.01), and 8- and 12-week groups (*P* < 0.01) were significant (Fig. 1A and B). In addition, a population of CD11b^−^ Ly6g^low^ cells/lymphocytes (CD45^+^) was approximately 0.38% to 6.76% in the blood, 0.17% to 0.64% in the spleen, and 2.50% to 40.10% in the liver of both the uninfected and infected mice. On the other hand, during the later stage of infection (after 8 weeks), the CD11b^+^ Ly6G^low^ population increased markedly and may represent either eosinophils or macrophages, as most were F4/80^+^ (data not shown) (21).

Given that the neutrophils were CD11b^+^ Ly6G^+^, similar to myeloid-derived suppressor cells (MDSCs), we cocultured CD3^+^ T cells labeled with carboxyfluorescein succinimidyl ester (CFSE) isolated from spleens of uninfected mice with neutrophils isolated from the spleens of mice which were 8 weeks postinfection with S. japonicum at a 1:1 ratio to determine whether the neutrophils could inhibit T cell proliferation (22). The neutrophils significantly reduced the number of proliferated CD4^+^ T cells (69.4% T cells alone versus 12.3% in the coculture; *P* < 0.001) and CD8^+^ T cells (68.6% in T cells alone versus 16.1% in coculture; *P* < 0.001) (Fig. 1C and D). Therefore, it is likely that the neutrophils expanding during S. japonicum infection in this model play an inhibitory role and block the function of T cells (23).

### Pathological changes in the liver caused by neutrophils.

The formation of egg granulomas in the liver is the primary pathology associated with S. japonicum infection in C57BL/6 mice (13). Therefore, we used immunofluorescence staining to describe the relationship between neutrophils and egg granulomas in the liver.

**FIG 2:**
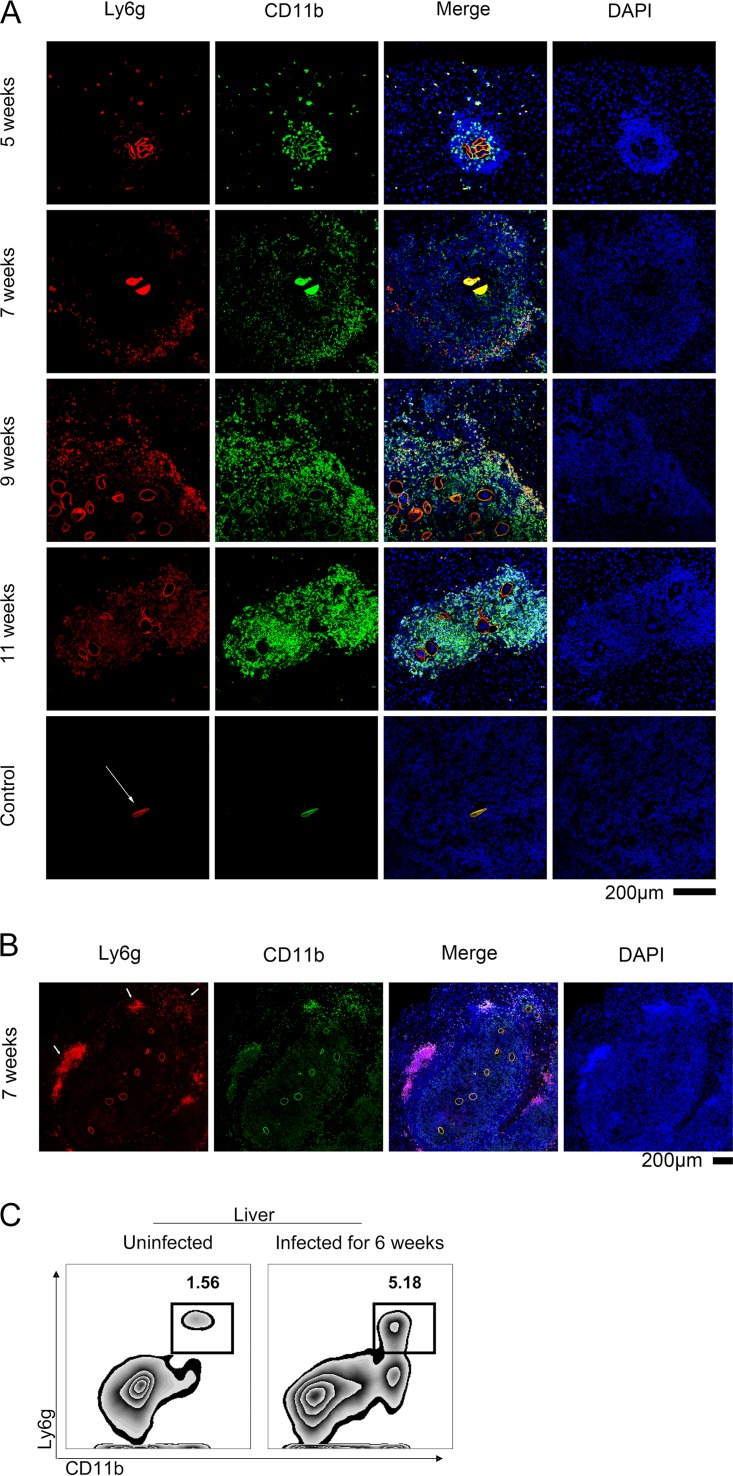
Pathological changes in the liver caused by neutrophils. (A) Liver tissue samples were obtained from C57BL/6 mice infected with S. japonicum at the indicated weeks postinfection and stained with PE-conjugated-anti-Ly6G and FITC-conjugated anti-CD11b antibodies. Representative images are shown. The white arrows indicate the shell of the egg, which is always brightly colored in red (ly6G^+^) and green (CD11b^+^) but is invisible in the blue channel. Scale bar, 200 μm. (B) A large granuloma and several eggs were observed in the liver 7 weeks postinfection. The arrows indicate neutrophils surrounding the new eggs and older neutrophils around the edge of the granuloma. Scale bar, 200 μm. (C) The percentage of neutrophils in the liver was assessed by flow cytometry. All of the control group consisted of unstained slides from WT mice infected with S. japonicum.

Seven weeks postinfection, neutrophils tended to cluster around the edge of the granuloma (Fig. 2A). By 9 weeks postinfection, the granulomas had enlarged and Ly6g^high^ neutrophils began to appear around the new eggs (Fig. 2A). Finally, during the late stage of the disease (11 weeks postinfection), the edges of the granuloma had blurred and the neutrophils were distributed throughout the liver (Fig. 2A). Interestingly, 8 weeks postinfection, the CD11b^+^ Ly6g^+^ neutrophils were observed situated around the edge of the granulomas in the liver (Fig. 2B). As in the flow cytometry analysis of the situation in the blood and spleen, a population of CD11b^−^ Ly6G^+^ cells was present in the liver regardless of infection status (Fig. 2C).

### Changes in the cytokine profile following S. japonicum infection.

**FIG 3:**
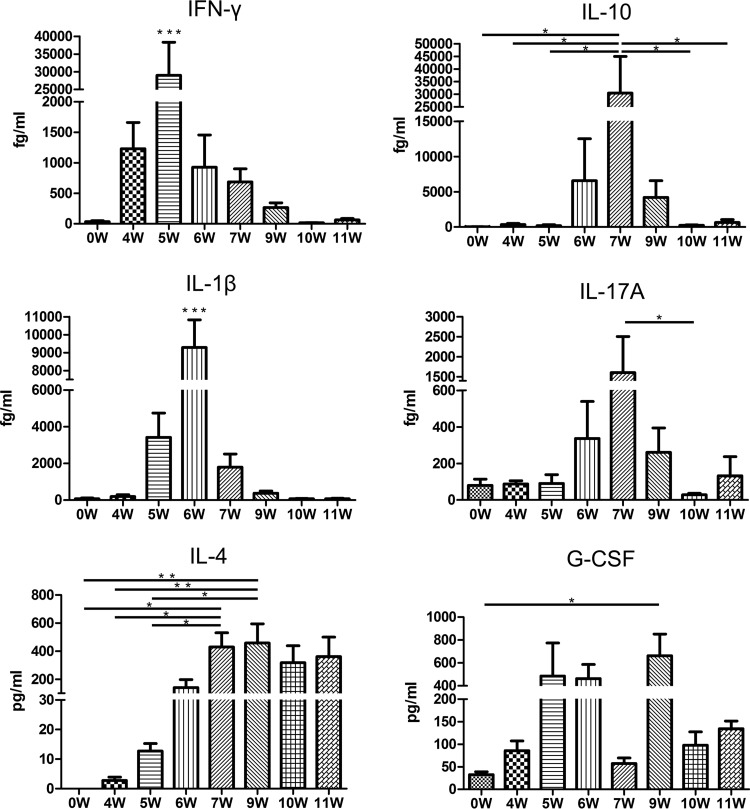
Changes in the cytokine profile following S. japonicum infection. Cytokine bead arrays were used to measure the serum levels of IL-17A, IFN-γ, IL-1β, and IL-10 in 6 mice/group infected with S. japonicum 4, 5, 6, 7, 8, 9, 10, and 11 weeks postinfection. *, *P* < 0.05; **, *P* < 0.01; ***, *P* < 0.001 (by repeated-measures ANOVA with Tukey's *post hoc* test).

Previous studies have shown that cytokines, particularly IL-17, may be associated with neutrophil infiltration into the liver (24). In addition, several studies have suggested roles for IFN-γ, IL-1β, IL-10, IL-4, and G-CSF in the pathogenesis of S. japonicum infection (13). Therefore, we used a cytokine bead array assay to measure the levels of IL-17, IFN-γ, IL-1β, IL-10, IL-4, and G-CSF in the serum over time (Fig. 3).

The first cytokine that increased was IFN-γ, which was elevated from 4 weeks postinfection, decreased after the fifth week, and was restored to normal levels after the ninth week. The difference between the groups was significant (*P* < 0.001). The peak point was approximately 28,988 fg/ml at 5 weeks postinfection and was significantly different from those of the other groups (*P* < 0.001).

IL-1β increased from 5 weeks postinfection, decreased after the sixth week, and returned to uninfected levels after the ninth week. The difference between the groups was significant (*P* < 0.001). The peak point was approximately 9,290 fg/ml at the sixth week after infection and was significantly different from those of other groups (*P* < 0.001).

IL-10 was elevated 6 weeks after infection, decreased after the seventh week, and returned to normal levels after the tenth week. The difference between the groups was significant (*P* < 0.01). The 7-week group (30,435 fg/ml) was significantly different from the other groups (*P* < 0.05), except for the 6-week (6,576 fg/ml) and 9-week (4,203 fg/ml) groups (*P* > 0.05).

IL-17 increased from 6 weeks postinfection, declined after the seventh week, and returned to uninfected levels after the ninth week. The difference between the groups was significant (*P* < 0.05). The difference between the 7 (1,606 fg/ml)- and 10 (28.8 fg/ml)-week groups was significant (*P* < 0.05).

IL-4 slowly increased from 5 weeks postinfection, maintaining a high level from the 7th week to the end of the observation period. The difference between the groups was significant (*P* < 0.001). The 7-week group (429 pg/ml) was significantly different from the uninfected (0 pg/ml), 4-week (2.8 pg/ml), and 5-week (12.8 pg/ml) groups (*P* value of <0.05 for each). The 9-week group was significantly different from the uninfected (*P* < 0.01), 4-week (*P* < 0.01), and 5-week (*P* < 0.05) groups, respectively.

G-CSF began increasing 4 weeks postinfection, severely declined at week 7, and then increased again during the ninth week. The difference between the groups was significant (*P* < 0.01). The difference between the uninfected (32.6 pg/ml) and 9-week (663 pg/ml) groups was significant (*P* < 0.05).

### The function of different subsets of γδ T cells declined postinfection.

γδ T cells have the capacity to produce a wide array of cytokines and function as an innate and adaptive immune cell, primarily in the mucosa and, to a lesser extent, in the blood and secondary lymphatic organs (25). Given that γδ T cells can produce all of the cytokines that were altered during S. japonicum infection, we next assessed whether the quantity of γδ T cells changed during S. japonicum infection in this model. However, there was no significant change in the proportion of the total γδ T cells/CD3^+^ T cells (data not shown).

**FIG 4:**
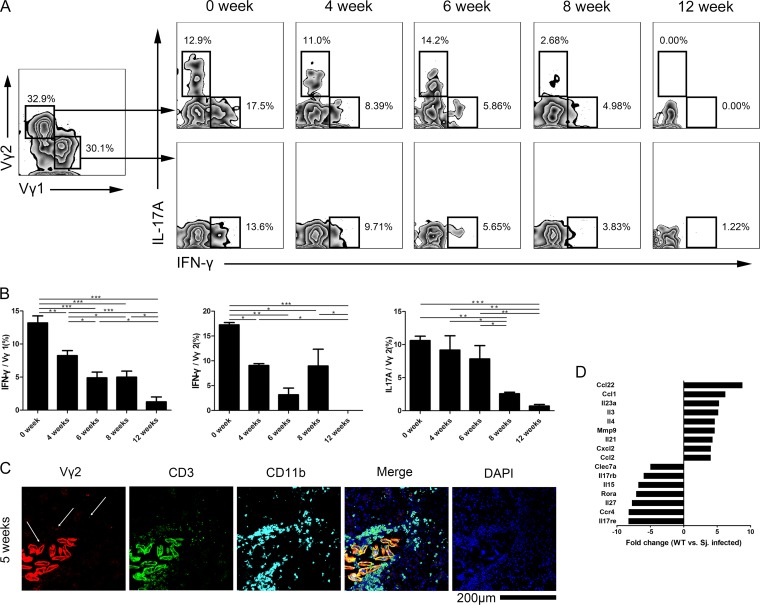
The functions of different subsets of γδ T cells declined postinfection. (A) Production of IL-17 and IFN-γ was assessed by intracellular flow cytometry in the Vγ1 and Vγ2 subsets of γδ T cells from spleen of C57BL/6 mice infected with S. japonicum. Representative plots are shown. (B) Summary graphs showing the change in expression of IL-17 and IFN-γ from Vγ1 and Vγ2 T cells collected from spleen of infected mice. *, *P* < 0.05; **, *P* < 0.01; ***, *P* < 0.001 (by one-way ANOVA with Tukey's *post hoc* test). (C) The fluorescence figures show the Vγ2 T cells located in the liver granuloma. The liver slide from C57BL/6 mice infected with S. japonicum for 5 weeks was stained with PerCp-efluor710-anti-Vγ2 TCR, FITC-anti-CD3, APC-anti-CD11b, and DAPI. The white arrow indicates the Vγ2 T cells in the granuloma. Scale bar, 200 μm. (D) γδ T cells from spleen of mice 6 weeks after infection with S. japonicum were analyzed with a real-time PCR array containing 84 different genes. Changes in gene expression greater than 3-fold are shown. Data are from three experiments. Three or four mice per group were used.

Therefore, we looked more closely at the function of the Vγ1 and Vγ2 T cell subsets to determine if functional changes were occurring. Both subsets produced IFN-γ; however, only the Vγ2 T cells produced IL-17 (Fig. 4A). Cytokine production from both the Vγ1 and Vγ2 T cell subsets decreased during the course of the infection (Fig. 4A and B). On the other hand, during observation of liver slices, the Vγ2 T cells were located beside the eggs or lying inside the granuloma (Fig. 4C).

The percentage of IL-17A^+^/Vγ2 T cells changed following infection (*P* < 0.001). The 8-week (2.6%) group was significantly different from the uninfected (10.6%), 4-week (9.2%), and 6-week (7.8%) groups (*P* < 0.05). The 12-week (0.7%) group was significantly different from the uninfected (*P* < 0.001), 4-week (*P* < 0.01), and 6-week (*P* < 0.01) groups.

The percentage of IFN-γ^+^/Vγ1 T cells varied during the course of the infection (*P* < 0.001). The uninfected group (13.2%) was significantly different from the 4-week (8.2%; *P* < 0.01), 6-week (4.9%; *P* < 0.001), 8-week (5.0%; *P* < 0.001), and 12-week (1.3%; *P* < 0.001) groups. The 4-week group was significantly different from the 6-week (4.9%; *P* < 0.05), 8-week (5.0%; *P* < 0.05), and 12-week (1.3%; *P* < 0.001) groups. The 12-week group was significantly different from the 6-week and 8-week groups (*P* < 0.05).

The percentage of IFN-γ^+^/Vγ2 T cells changed over time throughout the course of the infection (*P* < 0.001). The uninfected group (17.2%) was significantly different from the 4-week (9.1%; *P* < 0.05), 6-week (3.2%; *P* < 0.01), 8-week (9.0%; *P* < 0.05), and 12-week (0%; *P* < 0.0001) groups. The 12-week group was different from the 4-week and 8-week groups, respectively (*P* < 0.05).

A quantitative PCR (qPCR) array was performed to determine if other cytokines were also being produced by the γδ T cells. Following infection, expression levels of the immunomodulatory cytokines C-C motif chemokine 22 (CCL22), CCL1, IL-23α, IL-3, IL-4, matrix metalloproteinase 9 (MMP9), IL-21, C-X-C motif chemokine 2 (CXCL2), and CCL2 were increased. Levels of C-type lectin domain family 7 member A (CLEC7a), interleukin 17 receptor B (IL-17Rb), IL-15, retinoid-related orphan receptor alpha (RORα), IL-27, C-C chemokine receptor type 4 (CCR4), and interleukin 17 receptor E (IL-17re) were decreased in the γδ T cell subsets (Fig. 4D).

### The function of Vγ2 T cells *in vivo*.

**FIG 5:**
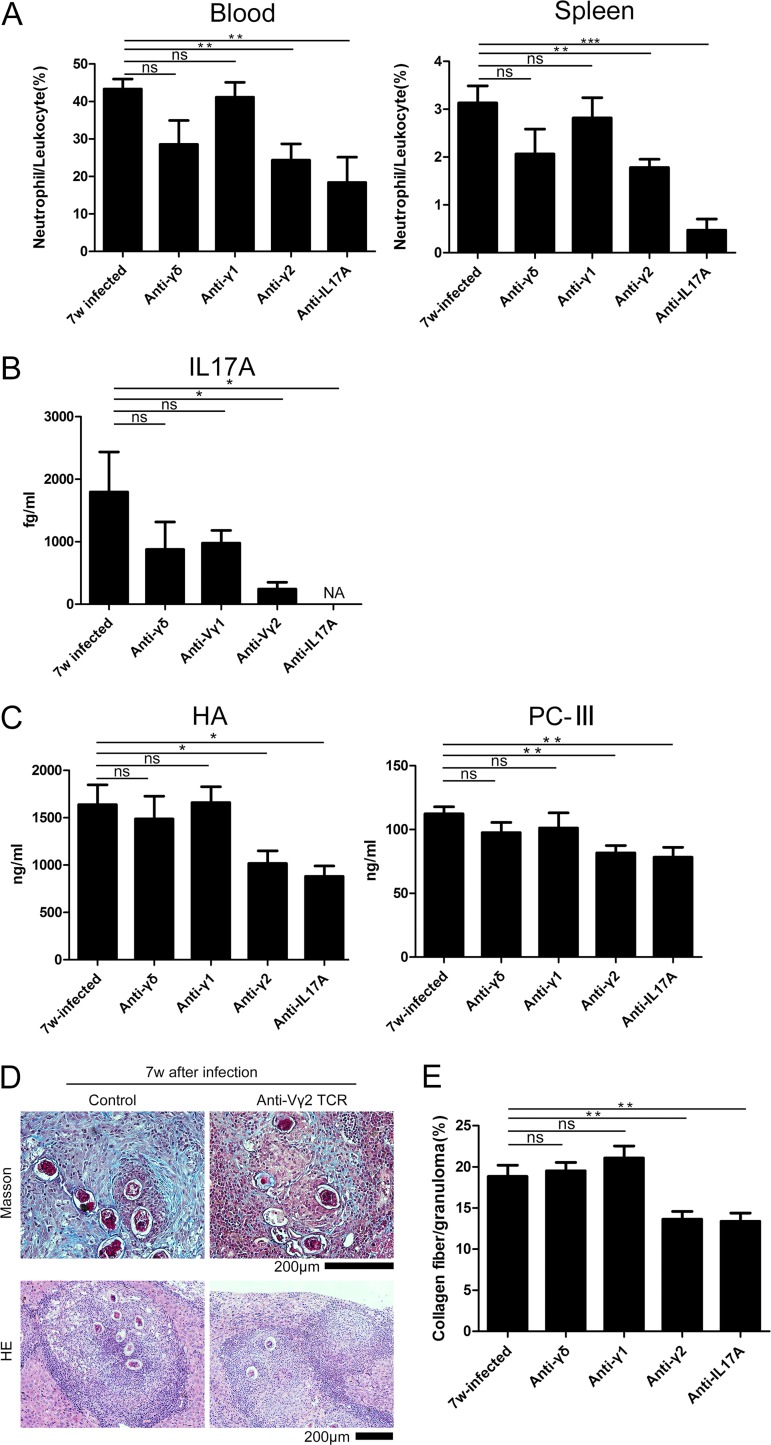
Depletion of Vγ2 T cells reduced the number of neutrophils in the blood and spleen. C57BL/6 mice were infected with S. japonicum (*n* = 5 mice/group), and then Vγ2 T cells were depleted using a monoclonal antibody 6 weeks postinfection. Liver, blood, and spleen samples were collected 8 days after depletion. (A) Summary graphs showing the percentages of neutrophils/CD45^+^ cells in the blood and spleen with and without depletion. (B) Summary graph showing the IL-17A level in serum with and without depletion. NA, not applicable. (C) Summary graph exhibiting the HA and PC-III levels in the serum with and without depletion. (D) Representative image of liver tissue stained with Masson's trichrome staining to measure fibrosis (blue). The image of the liver tissue stained with hematoxylin and eosin (HE) is shown. Scale bar, 200 μm. (E) Summary graph showing fibrosis as a percentage of the granuloma with or without depletion (number of granulomas observed in each group: 30, 28, 21, 44, and 30). ns, *P* > 0.05; *, *P* < 0.05; **, *P* < 0.01; ***, *P* < 0.001 (by Student's *t* test).

Previous studies have shown that γδ T cells are the major producers of IL-17A, rather than Th17 αβ T cells, and that IL-17A induces an inflammatory response in neutrophils (8, 26–28). To determine the relationship between γδ T cells and neutrophils during infection, we depleted γδ T cells from mice infected with S. japonicum from the fifth week after the parasites had laid eggs in the liver to the seventh week, when the level of IL-17A had increased. While there were no significant differences between the depleted and nondepleted groups (*P* > 0.05), there was a reduction in the average percentage of neutrophils/CD45^+^ cells (28.6% in the blood, 2.1% in the spleen) and the serum level of IL-17A (875 fg/ml) (Fig. 5A and B). When the Vγ2 T cells were specifically depleted, the percentage of neutrophils in the blood and spleen declined significantly, from 43.30% to 24.36% in the blood (*P* < 0.01) and 3.13% to 1.78% in the spleen (*P* < 0.01) (Fig. 5A). The percentages of neutrophils/CD45^+^ cells were significantly different when IL-17A was depleted, with an average level of 18.4% in the blood (*P* < 0.01) and 0.5% in the spleen (*P* < 0.001) (Fig. 5A). The absolute level of IL-17A in the serum was concurrently reduced from 1,797 fg/ml to 243 fg/ml (*P* < 0.05) (Fig. 5B). On the other hand, there was no significant difference after blocking the Vγ1 T cells regarding both the percentage of neutrophils/CD45^+^ cells and the level of IL-17A (*P* > 0.05).

The levels of hyaluronic acid (HA) and collagen type III (PC-III) in the serum were tested to analyze the degree of liver fibrosis. When the Vγ2 T cells were blocked, HA declined from 1,639 to 1,018 ng/ml (*P* < 0.05) and the PC-III level dropped from 112 to 81 ng/ml (*P* < 0.01). When IL-17A was depleted, HA declined to 880 ng/ml (*P* < 0.05) and the PC-III level dropped to 78 ng/ml (*P* < 0.01). There was no significant difference when the γδ T and Vγ2 T cells were depleted (*P* > 0.05) (Fig. 5C). The percentage of collagen fiber/granuloma in the liver was significantly reduced when the Vγ2 T cells (from 18.8% to 13.6%; *P* < 0.01) or IL-17A (from 18% to 13.4%; *P* < 0.01) was blocked (Fig. 5D and E). The difference in the anti-γδ T (19.5%) or anti-Vγ1 T (21.1%) cell group was not significant (*P* > 0.05).

### Adoptive transfer of γδ T cells to RAG^−/−^ mice prior to infection with S. japonicum.

**FIG 6:**
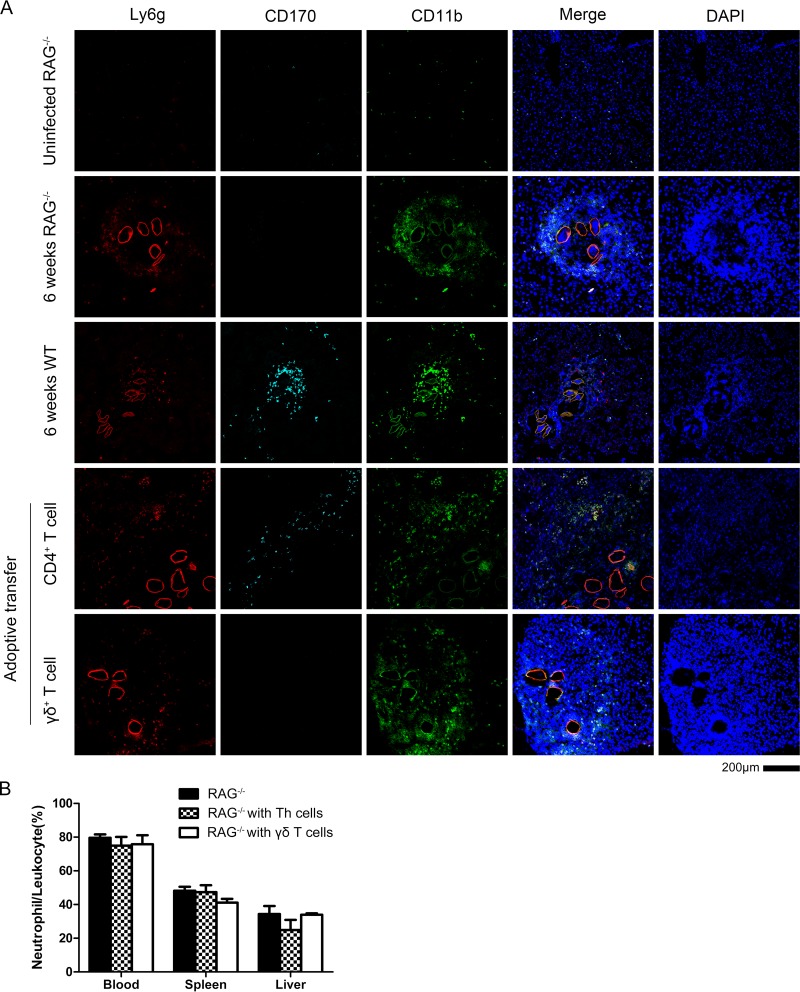
Adoptive transfer of γδ and CD4^+^ T cells to RAG^−/−^ mice following S. japonicum infection. (A) RAG^−/−^ mice (*n* = 3/group) were infected with S. japonicum, and 6 weeks later they received an adoptive transfer of either γδ T cells or CD4^+^ T cells isolated from WT mice infected with S. japonicum. Representative liver tissue images from the different groups stained with Ly6G, CD11b, CD170, and DAPI are shown. Images are from two experiments. Scale bar, 200 μm. (B) The percentage of neutrophils/CD45^+^ cells in the blood, spleen, and liver of RAG^−/−^ mice was assessed by flow cytometry.

To confirm the function of γδ T cells in mice with schistosomiasis, the adoptive transfer of the cells from the spleen of wild-type (WT) mice into RAG^−/−^ mice, which lacked mature T cells and B cells, was implemented. First, the RAG^−/−^ mice and the WT mice were infected with S. japonicum at the same time. As a result, there were lots of neutrophils around the eggs of S. japonicum in the center of the egg granuloma in the infected RAG^−/−^ mice (Fig. 6A). It is suggested that neutrophils are recruited by the eggs with or without the help of T/B cells in the RAG^−/−^ mice. Compared to the egg granuloma in liver of WT mice owning plenty of eosinophils (CD170^+^ cells), there were few eosinophils in the granuloma in liver of RAG^−/−^ mice during the early stage of infection (5 to 6 weeks).

Six weeks postinfection with S. japonicum, the γδ T cells and CD4^+^ T cells from spleen of infected WT mice were isolated and then transferred into different groups of infected RAG^−/−^ mice. One week after the adoptive transfer, none of the neutrophil proportions in the blood, spleen, or liver of RAG^−/−^ mice were increased compared to those of the nontransferred group (*P* > 0.05) (Fig. 6B).

On the other hand, in the group to which CD4^+^ T cells were transferred, the eosinophils appeared around the edge of egg granuloma in the liver of RAG^−/−^ mice. As shown in Fig. 6A, the eosinophils were newly recruited because they were not scattered in the egg granuloma, as is the case for WT mice. It is suggested that the recruitment of eosinophils is involved in the help of CD4^+^ T cells, while the γδ T cells could not have the same effect. The RAG^−/−^ mice began dying beginning at the eighth week postinfection with S. japonicum, and 2 out of 20 mice were still alive at the tenth week.

## DISCUSSION

The main focus of our study was to understand the factors driving neutrophil accumulation in the liver during S. japonicum infection, particularly in the context of γδ T cells. In our model, the number of neutrophils in the liver, blood, and spleen began to increase 6 weeks after infection and remained elevated through 12 weeks postinfection. Concurrent with the increase in neutrophils, IL-17 levels were increased. While the number of IL-17-producing Vγ2 T cells did not markedly increase following infection, we showed by depleting γδ T cells that they were helping to recruit neutrophils to granulomas caused by S. japonicum eggs and aggravating liver fibrosis. The serum levels of IL-17A and IFN-γ were also reduced in the γδ T cell-depleted mice. Interestingly, the ability of γδ T cells to produce cytokines (IL-17A and IFN-γ) decreased as the infection progressed. The γδ T cells may also be recruiting neutrophils by secreting CCL22, CCL1, IL-23α, IL-3, or IL-4.

While it is likely that γδ T cells contribute to the pathogenesis of schistosomiasis, it is also important to note that they were not necessary for granuloma formation, as mice depleted of γδ T cells formed granulomas. In addition, neutrophils were found around the liver granulomas when we depleted the Vγ2 TCR. There is also evidence that blocking IL-17A reduces the number of neutrophils present in the liver but does not eliminate them (1). On the other hand, the granuloma formation in the livers of RAG^−/−^ mice also suggests that the formation is dominated by innate immunity. Thus, it is likely that the presence of S. japonicum eggs recruits neutrophils to the liver with or without the help of T or B cells (29, 30).

Granulomas progress through several key stages as they mature. During the early stage of formation, the presence of parasite eggs rapidly recruits neutrophils that are similar to MDSCs in terms of phenotype and function (31, 32). During granuloma enlargement, neutrophils with a CD11b^+^ Ly6G^+^ phenotype were present at the border of the granulomas. Our results suggest that these neutrophils are distinct (high fluorescence) from the neutrophils in the middle of the granuloma (low fluorescence), suggesting they have a different function. In addition, eosinophils tended to be grouped toward the middle of the granuloma while the neutrophils were on the periphery, suggesting they have antagonistic functions. During late infection (>8 weeks postinfection), the CD4^+^ T cells, γδ T cells, and CD8^+^ T cells all tend to have reduced cytokine-producing abilities (33–37). Notably in our study, γδ T cells were no longer capable of producing IL-17A. These observations suggest a complex interaction whereby γδ T cells enhance granulation by producing IL-17A and recruiting neutrophils, which then inhibit T cell function (e.g., proliferation) (22, 38) and cause fibrosis, preventing granulation from continuing with unlimited expansion (39).

A subset of innate IL-17-producing γδ T cells is produced during fetal development (IL-1R^+^ IL-23R^+^ CCR6^+^), termed Tγδ17, which have an innate ability to make IL-17 (40, 41). In adults, Tγδ17 cells in the thymus preferentially express Vγ4 (42). Their ability to produce IL-17 is not related to TCR triggering but to the transcription factors SOX4, SOX13, TCF1, and LEF1. SOX4 and SOX13 directly regulate the two requisite Tγδ17 cell-specific genes *Rorc* and *Blk*. The TCF1 and LEF1 transcription factors counter the SOX proteins and induce expression of alternative effector genes. In addition, the development of natural Tγδ17 cells is also regulated by the transcription factor ETV5 (43, 44). Mature γδ T cells no longer require TCR stimulation to produce IL-17 and can produce the cytokine in response to IL-1 and IL-23 alone (17, 30). However, in this study, when we blocked the γδ TCR, the IL-17A level in the serum declined.

The Vγ1 and Vγ2 subsets of γδ T cells seem to have distinct roles in S. japonicum infection. The Vγ1 cells produce IFN-γ, which is an inflammatory cytokine that helps to recruit macrophages and eosinophils. In contrast, Vγ2 T cells produce both IL-17A and IFN-γ. IL-17A recruits neutrophils with an MDSC-like phenotype that inhibit inflammation. In previous studies, cells expressing Vγ1 have been shown to reduce bacterial clearance in a Listeria model, although γδ T cells as a whole promote clearance (3, 13). In this study, both subsets lost the ability to produce cytokine as the disease progressed. It is possible that IL-4 or other immunosuppressive cytokines have contributed to the loss of function by creating immunosuppressive microenvironments.

This is the first finding that subsets of γδ T cells play a role, albeit partial, in the pathological process of schistosome infection. The neutrophils comprised a large part of the cells forming granulomas in this model. However, our study is limited in the following ways: (i) we did not transfer the Vγ2 T cells to the RAG^−/−^ mice to demonstrate their function by acquisition of a trait; (ii) we did not measure the IL-17A in the serum after the cells were adoptively transferred to a RAG^−/−^ host; and (iii) fully defining the interaction between CD4^+^ T cells and CD170^+^ eosinophils requires further study.

## MATERIALS AND METHODS

### Ethics statement.

This study was carried out in strict accordance with the recommendations of the guide for the care and use of laboratory animals of the Ministry of Health, China. The protocol was approved by the Laboratory Animal Welfare & Ethics Committee (LAWEC), National Institute of Parasitic Diseases, Chinese Center for Diseases Control and Prevention (permit number IPD-2015-22).

### Animals.

C57BL/6 (WT) mice, 6 to 8 weeks of age, were purchased from Shanghai Laboratory Animal Center, Chinese Academy of Sciences. RAG1^−/−^ mice were obtained from the Model Animal Research Center of Nanjing University (China). All mice were maintained under specific-pathogen-free (SPF) conditions at the National Institute of Parasitic Diseases.

Cercariae were provided by the Key Laboratory of Parasite and Vector Biology, Ministry of Health (Shanghai, China). Each mouse was infected through the skin with 20 cercariae.

### Monoclonal antibody blocking experiment.

To deplete immune cells or neutralize cytokines, mice were injected through the tail vein with 50 μg twice weekly for γδ TCR (clone GL3; BD Biosciences), 50 μg twice weekly for Vγ2 TCR (clone UC3-10A6; BioXCell), 50 μg twice weekly for Vγ1 TCR (clone 2.11; BioXCell), and 25 μg three times weekly for anti-IL-17A (clone 17F3; BioXCell).

For the adoptive transfer assay, the CD3^+^ cells from spleen of WT mouse infected for 4 weeks with S. japonicum were purified by a magnetic column (Miltenyi Biotec, Bergisch Gladbach, Germany). The CD4^+^ T cells and γδ T cells were isolated by BD FACSAria III flow cytometry and subjected to CD4^+^ or γδ TCR^+^ labeling. The CD4^+^ T cells and γδ T cells (2 × 10^5^) were injected through the tail vein into each RAG1^−/−^ mouse.

### Flow cytometry.

Blood samples were collected in tubes containing heparin. Spleen samples were mashed through a 70-mm cell strainer. Liver samples were incubated in collagenase IV for 30 min, mashed through a 70-mm cell strainer, and then separated from hepatocytes by centrifugal mixing with 35% isotonic Percoll. All single-cell suspensions were treated with NH_4_Cl erythrocyte lysis buffer. Cells were stained with directly conjugated antibodies (listed below) for 30 min at 4°C in the dark in phosphate-buffered saline–1% bovine serum albumin (PBS–1% BSA). Fixable viability dye eFluor 780 (1:1,000; eBioscience, San Diego, CA, USA) was added to exclude dead cells. For intracellular staining, single-cell suspensions were stimulated in Dulbecco's modified Eagle's medium (DMEM) containing 10% fetal bovine serum (FBS), 100 IU/ml penicillin, 100 mg/ml streptomycin, 0.5% β-mercaptoethanol, 81 nM phorbol myristate acetate, 1.34 μM ionomycin, and 10.6 μM brefeldin A (eBioscience) for 4 h at 37°C. Surface antigens were stained first, followed by fixation and permeabilization using the Foxp3 staining buffer set (eBioscience) and then staining of intracellular proteins. All experiments were performed using a Cyan ADP flow cytometer with Submit software. Data analyses used FlowJo software, version 9.7.1.

All antibodies were purchased from eBioscience, except that Ly6G and IFN-γ were from BD Biosciences (San Diego CA, USA), Vγ1 was from BioLegend (San Diego CA, USA), CCR2 was from R&D Systems (Minneapolis, MN, USA), and CD45 was from Life Technologies (Gaithersburg, MD, USA). The following antibodies were used in the γδT cell phenotyping panel: CD45-Pacific Orange (1:100; clone 30-F11), CD11b-allophycocyanin (APC)-eFluor 780 (1:400; clone M1/70), CD4-APC-eFluor 780 (1:400; clone GK1.5), γδ TCR-phycoerythrin (PE) (1:200; clone GL3), CD19-APC-eFluor 780 (1:400; clone eBio1D3), CD27-PE-Cy7 (1:100; clone LG.7F9), Vγ1-fluorescein isothiocyanate (FITC) (1:100; clone 2.11), Vγ2-peridinin chlorophyll protein (PerCP)-eFluor 710 (1:100; clone UC3-10A6), IFN-γ-brilliant violet 421 (1:200; clone XMG1.2), IL-17A–APC (1:200; clone eBio17B7), and fixable viability dye eFluor 780. The following antibodies were used for the neutrophil panel: CD45-Pacific Orange (1:100; clone 30-F11), CD11b-FITC (1:200; clone M1/70), Ly6G-PE-CF594 (1:200; clone 1A8), Ly6C-PE-Cy7 (1:400; clone HK1.4), F4/80-APC(1:200; clone BM8), cKIT-eFluor 450 (1:400; clone 2B8), CCR2-PE (1:50; clone 475301), CXCR4-PerCP-eFluor 710 (1:100; clone 2B11), and fixable viability dye eFluor 780.

### Immunofluorescence.

Tissues were embedded in optimal cutting temperature compound (OCT), frozen in liquid nitrogen, and then cut into 6-μm pieces. Tissue on the plate was washed three times with PBS, fixed in acetone for 15 min, and then washed three times with PBS. The tissue was then incubated in blocking buffer (10% goat serum, 5% BSA) for 1 h at 25°C prior to addition of mixed monoclonal antibody culture supernatant at a 1:50 dilution at 4°C overnight. The antibodies then were removed by repeated washing in PBS, and the coverslips were mounted on 10 μl of mounting liquid (including 4′,6-diamidino-2-phenylindole [DAPI]). Ly6G-PE-CF594 and CD11b-FITC were used for neutrophils. Images were taken on a confocal microscope (A1; Nikon) using the provided software (NIS-Elements).

### Masson's trichrome staining.

Fresh liver tissues (round pieces about 7 mm in diameter cut from the edge of the liver) were fixed in 4% formaldehyde overnight and routinely paraffin embedded. Paraffin sections (5 μm) were prepared from each liver tissue sample. The liver tissue sections were stained by Masson's trichrome staining to evaluate collagen content and distribution. The collagen fibers were stained blue, cell nuclei were stained black, and the background was stained red. Each stained section was examined by optical microscopy with ×200 magnification and identical settings. Thirty pictures were taken from three sections from each tissue, which included the egg granulomas in the center. The percentage of granuloma areas with collagen-positive color (blue), i.e., the positive blue color area/granuloma area (measured as a percentage), was analyzed using Image-Pro Plus 6.0 software. Every picture was evaluated in double-blind fashion by two independent investigators.

### T cell proliferation assay.

Spleen neutrophils from S. japonicum-infected mice and splenic CD3^+^ T cells from WT mice were isolated with a magnetic column (Miltenyi). CD3^+^ T cells were labeled with CellTrace CFSE (carboxyfluorescein succinimidyl ester) by following the manufacturer's instructions (Invitrogen, Carlsbad, CA). Equal numbers of cells (4 × 10^5^) were cocultured in a 96-well U-bottom plate. CD3/CD28 Dynabeads (Invitrogen) were added according to the manufacturer's instructions. After 48 h, T cell proliferation was evaluated on a cyan ADP flow cytometer using Summit software with the following antibodies: CD8a-APC (1:600; clone 53-6.7), CD4-APC-eFluor 780 (1:400; clone GK1.5), Ly6G-brillant violet 421 (1:400; clone 1A8), and 7-aminoactinomycin. Data analyses used FlowJo software, version 9.7.1.

### Cytokine analysis.

Cytometric bead array was performed according to the manufacturer's instructions to detect the serum levels of IFN, IL-10, IL-1β, IL-17A, IL-4, and G-CSF at different infection stages.

HA was measured using an enzyme-linked immunosorbent assay (ELISA) kit purchased from R&D Systems (Minneapolis, MN, USA). PC-III was detected by an ELISA kit obtained from Jiancheng Bioengineering Institute (Nanjing, Jiangsu, China).

### Real-time PCR.

Total RNA was extracted from fresh liver tissue homogenized in TRIzol reagent (Invitrogen) according to the manufacturer's protocol. RNA purity and concentration were assessed by spectrophotometry. Reverse transcriptase (RT) reactions for cDNA synthesis were performed using PrimeScript RT master mix (TaKaRa Bio, Tokyo, Japan). The relative mRNA expression level was determined by real-time quantitative PCR (qPCR) with a SYBR green I PCR master (TaKaRa) kit on an ABI ViiATM7 machine according to the manufacturer's protocol.

For the qPCR array assay, γδ T cells were isolated using a BD FACSAria III flow cytometer with CD3^+^ γδ TCR^+^ labeling. The purity of isolated γδ T cells was validated by flow cytometry. Since the γδ T cells are only a small part of whole white blood, cells from spleens of 10 normal mice and 4 infected mice were used for each sample.

### Statistics.

All of the data were analyzed using SPSS 13.0 and GraphPad Prism, version 5, using one-way analysis of variance, except for the blocking tests, which used a *t* test, with a *P* value of <0.05 indicating significance.
